# Evaluating the Color of Chocolate Chip Cookies: Comparative Performance of Instrumental Colorimetry, Digital Image Analysis, and Computer Vision Relative to Human Perception

**DOI:** 10.1111/1750-3841.71133

**Published:** 2026-05-11

**Authors:** Natalia Calderón, Eniola Ola, Han‐Seok Seo

**Affiliations:** ^1^ Department of Food Science University of Arkansas Fayetteville Arkansas USA

**Keywords:** color, colorimeter, computer vision, cookie, image analysis, perception, sensory

## Abstract

Food color is commonly quantified using colorimeters; however, these instruments may be impractical in home‐based or small‐scale production settings. More accessible alternatives, such as digital image analysis and computer vision, may offer reliable color quantification when conventional instrumentation is unavailable. The objective of this study was to identify the color‐measurement method and sampling strategy that most accurately and reliably predict consumers’ perceived cookie color. Three approaches to characterizing the color of chocolate chip cookies were compared and evaluated for concordance with human perception: instrumental colorimetry (HunterLab colorimeter), digital image analysis (Adobe Photoshop), and computer‐vision analysis (OpenCV/Python). One hundred consumers evaluated the perceived lightness, redness, and yellowness of 15 cookie samples (3 cookies × 5 commercial brands) using 15‐cm line scales designed to approximate L*, a*, and b* color constructs. Instrumental and image‐based measurements were collected under two sampling conditions: (1) four standardized surface locations on each cookie (selected point) and (2) whole‐surface averaging (whole cookie). Across methods, selected‐point sampling generally produced higher L* values than whole‐cookie sampling. Multivariate analyses showed that all methods differentiated among brands. However, agreement with sensory configurations was strongest for instrumental colorimetry under selected‐point sampling and for computer vision under whole‐cookie sampling. Predictive modeling further indicated that selected‐point instrumental colorimetry most consistently predicted perceived lightness and yellowness, whereas whole‐cookie computer‐vision generated the best‐performing predictions of perceived redness. In conclusion, selected‐point colorimetry showed the closest correspondence with perceived cookie color in this dataset, while computer vision demonstrated strong promise as a practical alternative for color quantification in resource‐limited settings.

## 1 Introduction

Food appearance strongly shapes consumers’ first impressions and expectations of product quality (Altmann et al. 2023; Ola and Seo 2025). Among appearance‐related attributes such as color, shape, geometry, gloss, size, visual texture, surface properties, and opacity, color is often one of the most immediate and influential cues in food evaluation and acceptance (Clydesdale 1991; Kane et al. 2003). Before consumers assess flavor or texture, they often use color to infer freshness, ingredient composition, processing conditions, flavor expectations, safety, and overall acceptability (Clydesdale 1991; Yang et al. 2016; Jeesan and Seo 2020; Wang et al. 2024; Wang et al. 2025). Because of its strong influence on consumer expectations and acceptance, accurate color measurement is important in food quality evaluation, product development, and sensory research.

Color is the perceptual response that occurs when light reflected from an object is processed by the human visual system. In food research, perceived color is commonly described using three perceptual dimensions: lightness, hue, and chroma (Chae 2022). Lightness refers to the perceived brightness of a surface relative to a reference white under the same illumination and is closely related to surface reflectance properties (Corney et al. 2009; Chae 2022). Hue distinguishes color categories such as red, green, and blue, and is often represented as an angular component in many color systems (Corney et al. 2009). Chroma describes the vividness or saturation of a hue and reflects the perceived intensity or purity of the color (Corney et al. 2009; Hasenbeck et al. 2014; Chae 2022). Together, these dimensions provide a useful framework for describing how consumers perceive food color.

Traditionally, food color has often been evaluated visually, especially in sensory evaluation contexts. Although visual assessment is simple and inexpensive, it is affected by lighting conditions, background contrast, food morphology, and inter‐assessor variability, all of which can reduce consistency and repeatability (Meléndez‐Martínez et al. 2005; Hasenbeck et al. 2014; Seo et al. 2020; Wang et al. 2024). To address these limitations, the food industry has widely adopted instrumental methods such as colorimeters, which provide more objective and standardized color measurements (Afshari‐Jouybari and Farahnaky 2011; Sun et al. 2016). More recently, image‐based analysis using programming environments (e.g., Python) and computer‐vision libraries (e.g., OpenCV) has emerged as another promising approach for automated food color evaluation from digital images (Raguraman et al. 2021).

Colorimeters measure reflected or transmitted light within the visible spectrum and express results in standardized color spaces designed to approximate human perception (Macdougall 2010; Choduhurry 2014). Common instruments include Minolta chroma meters, HunterLab colorimeters, and Dr. Lange colorimeters (León et al. 2006). In food science, color is frequently reported in the CIELAB (CIE L*, a*, b*) color space, where L* denotes lightness (0 = black; 100 = diffuse white), a* represents the red‐green axis (positive values = red; negative values = green), and b* indicates the yellow‐blue axis (positive values = yellow; negative values = blue) (Afsghari‐Jouybari and Farahnaky 2011). In parallel, computer vision systems typically include a camera, controlled illumination, an imaging chamber, and image‐processing software, enabling non‐contact color measurement from digital images (Pedreschi et al. 2006; Afsghari‐Jouybari and Farahnaky 2011; Sun et al. 2016; Goñi and Salvadori 2017). Because digital images are captured in RGB format (red, green, and blue), these data can be converted into CIELAB coordinates to facilitate comparison with conventional colorimeter outputs (Yam and Papadakis 2004; Afsghari‐Jouybari and Farahnaky 2011; Goñi and Salvadori 2017).

Previous studies have compared colorimeter‐based and digital image‐based measurements across a wide range of foods, including meat (O'Sullivan et al. 2003; Sun et al. 2016; Goñi and Salvadori 2017; Tomasevic et al. 2019), fishes (Yagiz et al. 2009), vegetables (Goñi and Salvadori 2017), fruits (Afshari‐Jouybari and Farahnaky 2011; Goñi and Salvadori 2017; Sabat et al. 2024), bakery products (Kane et al. 2003; Goñi and Salvadori 2017), potato chips (Pedreschi et al. 2006), pizzas (Yam and Papadakis 2004), and dairy products (Minz and Saini 2021). These studies suggest that image‐based methods can serve as useful and efficient alternatives to conventional instrumental measurements. However, an important gap remains. Relatively few studies have directly examined which instrumental approach best reflects human perception of color, rather than simply comparing agreement between instruments (Segnini et al. 1999; Kane et al. 2003; Quevedo et al. 2010). In practice, a method may be precise and objective but still fails to represent how consumers visually judge a product.

A key methodological distinction between colorimeters and image‐based systems is sampling coverage. Colorimeters generally measure color from a small, localized surface area, often approximately 2–5 cm^2^ (Girolami et al. 2013; Sabat et al. 2024), whereas image‐based methods can quantify color across all visible pixels of a sample. This difference may be especially important for foods with visually heterogeneous surfaces, where localized measurement may not fully represent the appearance consumers actually perceive. Despite this practical relevance, limited research has systematically examined how different sampling strategies within image‐based measurement influence correspondence with consumer perception.

Accordingly, the present study investigated which measurement approach and sampling strategy most accurately and reliably predict consumers’ perceived color of chocolate chip cookies. The novelty of this study lies in linking consumer sensory judgments to both instrumental modality and sampling strategy, rather than evaluating instrumental outputs alone. Specifically, we compared conventional colorimeter measurements with image‐based color analysis and further contrasted selected‐point sampling, intended to approximate localized colorimeter assessment, with whole‐cookie averaging, which captures the entire visible surface. This design allowed us to determine not only whether image‐based methods can better represent perceived color but also whether broader spatial sampling offers an advantage for visually non‐uniform foods.

Chocolate chip cookies were selected as the model product because they exhibit substantial visual heterogeneity due to irregular distributions of chocolate chips and localized browning (Frauenhoffer et al. 2025). This surface complexity makes them an appropriate test case for evaluating whether the measurement method and sampling extent influence the prediction of perceived color. By identifying the instrumental strategy that most closely aligns with consumer perception, this study provides practical insight into food color evaluation and contributes to the development of measurement approaches that are both objective and perceptually meaningful.

## 2 Materials and Methods

### 2.1 Participants

One hundred individuals (49 males and 51 females) with an age range between 18 and 80 years [mean age ± standard deviation (SD) = 37 ± 12 years] participated in this study. All participants reported no clinical history of major disease and reported consuming and purchasing cookies at least once per month. The study was conducted in accordance with the Declaration of Helsinki. The protocol (2311502798) was approved by the University of Arkansas Institutional Review Board (Fayetteville, AR, USA). All participants provided written informed consent prior to participation.

### 2.2 Cookie Samples and Imaging Setup

Fifteen chocolate chip cookies representing five commercial brands (three cookies per brand) were purchased from a local grocery store (Fayetteville, AR, USA). Images were acquired using a portable tabletop photobox (SAMTIAN photo light box; internal dimensions approximately 43.5 cm × 43.5 cm × 43.5 cm) designed to provide uniform diffuse illumination and to minimize ambient‐light interference. The enclosures use reflective inner walls to promote even light distribution and include integrated LED lighting (84 LEDs; correlated color temperature: 5500 K; Color Rendering Index ≥ 95) with an inline dimmer to adjust intensity. A Samsung Galaxy A54 5G smartphone camera (Samsung, Suwon, South Korea; triple camera system: 50 MP wide, f/1.8; 12 MP ultra‐wide, f/2.2; 5 MP macro, f/2.4) was mounted at the front opening of the photobox and positioned horizontally at a fixed distance of 17 cm from the cookie surface (camera optimal axis centered on the sample). Each cookie was placed at the center of the imaging area to standardize framing and minimize vignetting and edge shading. The photobox openings remained closed, except for the camera aperture, to reduce stray light and reflections during the image capture process.

### 2.3 Human Sensory Evaluation

One hundred participants evaluated the overall color of each whole‐cookie sample in terms of perceived lightness, redness, and yellowness using 15‐cm line scales anchored from 0 (“not at all”) to 100 (“extremely high”). The scales were designed to approximate the L*, a*, and b* constructs commonly reported in the CIELAB system. The 15 samples were presented in a single session according to a Williams Latin square design (Williams 1949). Sensory data were collected using Compusense Cloud (Compusense Inc., Guelph, ON, Canada). Upon completion of the sensory evaluation, each participant received a $10 e‐gift card as compensation.

### 2.4 Instrumental Colorimetry Measurement

Cookie color was measured using a portable colorimeter (MiniScan XE Plus, HunterLab, Reston, VA, USA), and results were reported as CIELAB values (L* = lightness; a* = red‐green axis; b* = yellow‐blue axis). Measurements were conducted under two sampling conditions: (1) “selected‐point” sampling, in which color was measured at four standardized surface locations (top, bottom, left, and right) on intact cookies, and (2) “whole‐cookie” sampling, in which cookies were crushed to create a more uniform sample matrix, after which measurements were taken at the same four positions. Although the term “whole‐cookie” sampling does not strictly correspond to this crushed‐sample preparation, it was retained in this study to maintain consistency with the sampling terminology used for the other analytical methods.

### 2.5 Digital Image Analysis

**FIGURE 1:**
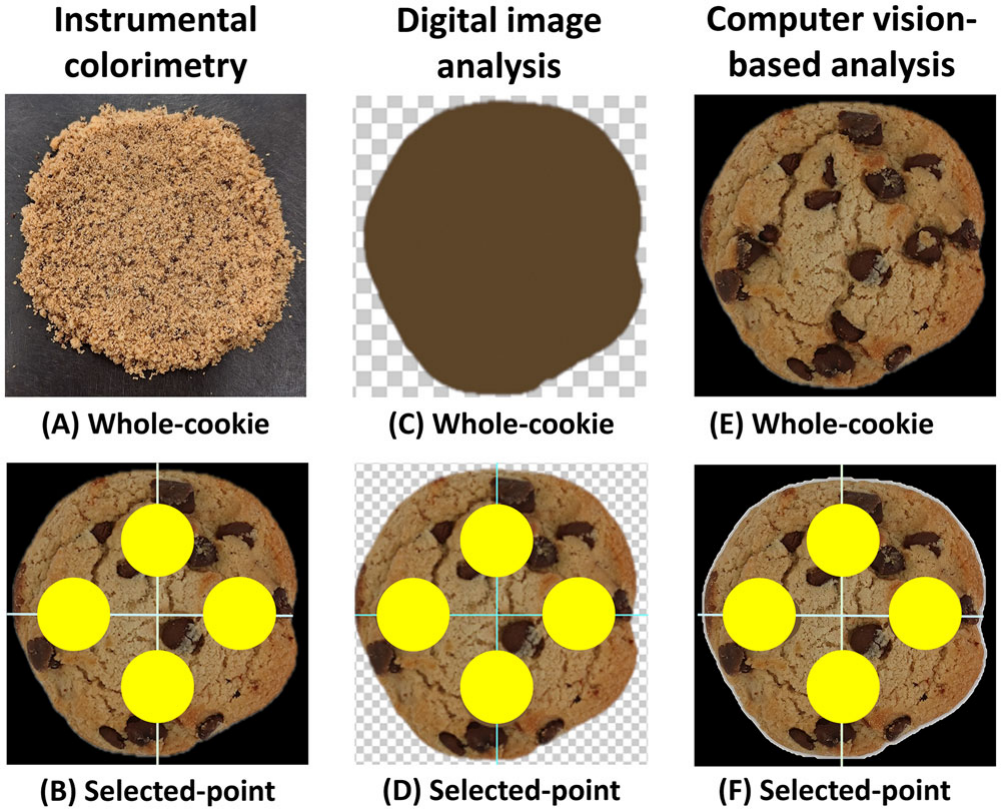
Examples of whole‐cookie (A,C,E) and selected‐point (B,D,F) sampling conditions for the three analytical methods: instrumental colorimetry, digital image analysis, and computer vision‐based analysis. Under selected‐point sampling, yellow circles indicate the locations measured by the colorimeter and the 300‐pixel‐diameter regions used for digital image analysis and computer vision‐based analysis.

Cookie images were analyzed in Adobe Photoshop (Adobe Systems, Inc., San Jose, CA, USA) to obtain CIE L*, a*, and b* values under two conditions. For “whole‐cookie” sampling, the cookie was segmented, and the mean color was calculated across all included pixels. For “selected‐point” sampling, fixed horizontal and vertical axes were drawn through the cookie center, and circular regions of interest (300‐pixel diameter) were placed at the top, bottom, left, and right positions (Figure 1). The mean color within each region was then calculated.

### 2.6 Computer Vision Analysis

Computer vision analyses were performed in Python 3.13.1 using OpenCV (Python Software Foundation, Fredericksburg, VA, USA). For “whole‐cookie” sampling, the background was removed, and the mean RGB values of all cookie pixels were calculated and converted to CIELAB coordinates. For “selected‐point” sampling, the cookie center was identified, four axis‐based locations (top, bottom, left, and right) were defined, circular regions (300‐pixel diameter) were extracted, and the mean RGB values within each circular region were calculated and converted to CIELAB coordinates.

### 2.7 Statistical Analysis

Statistical analyses were conducted using JMP Pro (version 18, SAS Institute Inc., Cary, NC, USA) and XLSTAT (Lumivero, Denver, CO, USA). To evaluate the effect of sampling condition (i.e., “whole‐cookie” vs. “selected‐point”), color parameters (L*, a*, and b*) obtained from each method across the 15 cookie samples were compared using Student's *t*‐tests. For each color parameter, differences among method conditions (i.e., instrumental colorimetry, digital image analysis, and computer vision‐based analysis) were assessed using one‐way analysis of variance (ANOVA).

Brand discrimination was evaluated using one‐way multivariate analysis of variance (MANOVA), with “brand” treated as a fixed effect. When MANOVA was significant, follow‐up one‐way ANOVAs were conducted, and mean separations were performed using Tukey's honestly significant difference (HSD) *post hoc* test. A statistical significance was set at *p* < 0.05.

Multivariate configurations (i.e., factor score matrices) derived from principal component analysis (PCA) based on lightness (L*), redness (a*), and yellowness (b*) were compared between sensory and analytical methods using the regression vector (RV) coefficient (Schlich 1996). RV values closer to 1 indicate greater similarity between the two configurations (Chapko and Seo 2019). Agglomerative hierarchical clustering (AHC) was conducted using Euclidean distance and Ward's linkage method to visualize clustering patterns among cookie brands. Clustering patterns were then compared across sensory and analytical methods.

Regression analyses were used to examine the relationships between predictor variables and sensory ratings and to identify the model that best fits the observed sensory responses. Linear and polynomial regression models (linear, quadratic, cubic, quartic, and quintic) were used to assess linear and curvilinear relationships, while a non‐linear power model was used to assess non‐linear relationships. The dataset was arranged as a matrix, with rows representing cookie samples and columns representing predictor variables and sensory ratings. Model performance was evaluated using the coefficient of determination (*R* ^2^), root mean square error (RMSE), corrected Akaike information criterion (AICc), and Bayesian information criterion (BIC). These metrics are widely used to evaluate and compare the predictive performance of regression models (Fahrmeir et al. 2021). Because no single model consistently performed best across all evaluation criteria, the best‐fitting model was selected based on the overall pattern of performance, with preference given to models showing higher *R* ^2^ and lower RMSE, AICc, and BIC values.

## 3 Results

### 3.1 Effects of Method and Sampling Condition on Color Parameters

**FIGURE 2:**
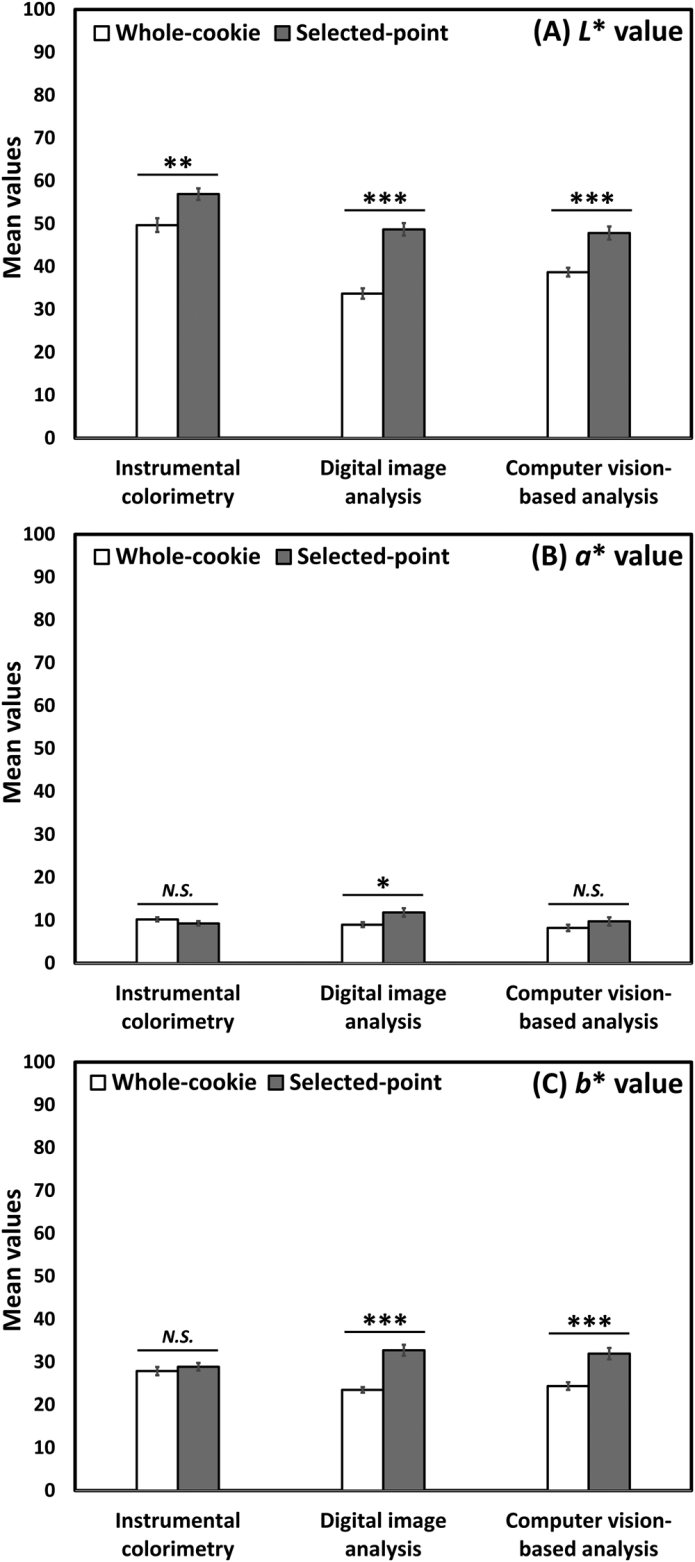
Mean comparisons between whole‐cookie and selected‐point sampling conditions for (A) L*, (B) a*, and (C) b* values across analytical methods. *, **, and *** indicate significant differences at *p* < 0.05, 0.01, 0.001, respectively. *N.S*. indicates no significant difference (*p* ≥ 0.05).

As shown in Figure 2, lightness (L*) values were higher under selected‐point sampling than under whole‐cookie sampling for all three analytical approaches: instrumental colorimetry (*t* = 3.42, *p* = 0.002), digital image analysis (*t* = 7.82, *p* < 0.001), and computer vision‐based analysis (*t* = 4.98, *p* < 0.001). For redness (a*), selected‐point sampling yielded higher values than whole‐cookie sampling only for digital image analysis (*t* = 2.48, *p* = 0.02); no significant sampling effect was observed for instrumental colorimetry (*t* = ‐1.34, *p* = 0.19) or computer vision analysis (*t* = 1.25, *p* = 0.22). For yellowness (b*), selected‐point sampling produced higher values than whole‐cookie sampling in digital image analysis (*t* = 6.46, *p* < 0.001) and computer vision analysis (*t* = 4.75, *p* < 0.001), but not in instrumental colorimetry (*t* = 0.76, *p* = 0.46).

**FIGURE 3:**
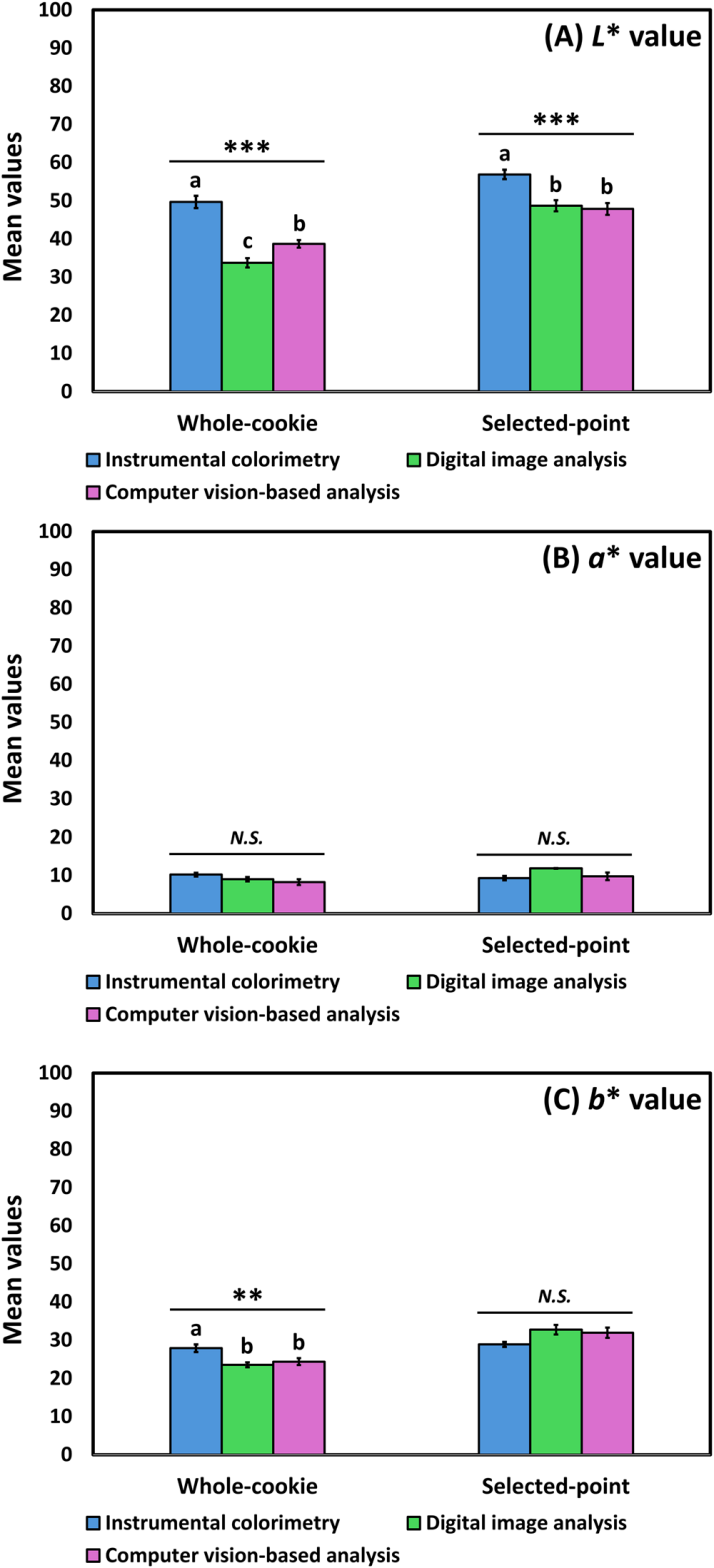
Mean comparisons among instrumental colorimetry, digital image analysis, and computer vision‐based analysis for (A) L*, (B) a*, and (C) b* values across whole‐cookie and selected‐point sampling conditions. ** and *** indicate significant differences at *p* < 0.01 and 0.001, respectively. Mean values with different letters represent a significant difference at *p* < 0.05. *N.S*. indicates no significant difference (*p* ≥ 0.05).

Under whole‐cookie sampling, instrumental colorimetry produced the highest L* values, followed by digital image analysis and computer vision (*F* = 39.07, *p* < 0.001; Figure 3). Under selected‐point sampling, instrumental colorimetry again produced the highest L* values (*F* = 11.77, *p* < 0.001), while digital image analysis and computer vision analysis did not differ significantly from one another. Instrumental colorimetry under whole‐cooking sampling also produced higher b* values than digital image and computer vision analyses (*F* = 7.42, *p* = 0.001), whereas differences among methods under selected‐point sampling were not significant (*F* = 2.97, *p* = 0.06). No significant differences among the three analytical approaches were observed for redness (a*) under either whole‐cookie sampling (*F* = 2.75, *p* = 0.08) or selected‐point sampling (*F* = 2.53, *p* = 0.09; Figure 3).

### 3.2 Brand Discrimination Across Sensory and Analytical Methods

Participants significantly discriminated among the five brands on the basis of perceived color (MANOVA: Wilks' Lambda = 0.004, *p* < 0.001). Significant brand effects were also detected for instrumental colorimetry, digital image analysis, and computer vision‐based analysis under both sampling conditions. Instrumental colorimetry showed strong discriminatory ability under whole‐cookie sampling (Wilks’ Lambda = 0.003, *p* < 0.001) and selected‐point sampling (Wilks’ Lambda = 0.001, *p* < 0.001). Digital image analysis also detected significant brand differences under whole‐cookie sampling (Wilks’ Lambda = 0.08, *p* = 0.02) and selected‐point sampling (Wilks’ Lambda = 0.004, *p* < 0.001). Similarly, computer vision analysis identified significant brand differences under whole‐cookie sampling (Wilks’ Lambda = 0.002, *p* < 0.001) and selected‐point sampling (Wilks’ Lambda = 0.003, *p* < 0.001). Together, these findings indicate that both human sensory evaluation and all analytical approaches reliably captured brand‐level differences in cookie color.

Table 1 summarizes the mean comparisons for lightness (L*), redness (a*), and yellowness (b*) across brands. For lightness (L*), brand effects were significant for sensory ratings (*p* < 0.001) and instrumental colorimetry under both sampling conditions (*p* < 0.001). Brand C was consistently the lightest, whereas Brand E was the darkest. *Post hoc* comparisons for selected‐point instrumental colorimetry closely matched the sensory pattern: Brand C > Brand A = Brand B = Brand D > Brand E. Digital image analysis and computer vision analysis detected significant differences under whole‐cookie sampling (*p* < 0.05), but not consistently under selected‐point sampling; nevertheless, Brand C and Brand E remained the lightest and darkest, respectively, across approaches.

**TABLE 1:** Mean (± standard deviation) color‐parameter values for five cookie brands across measurement and sampling conditions.

Color parameter	Measurement condition	Sampling condition	Brand A	Brand B	Brand C	Brand D	Brand E	*F* ratio (*p* value)
Lightness (L*)	Human sensory evaluation		47.15^b^ (± 5.48)	38.43^b^ (± 8.24)	78.22^a^ (± 3.71)	37.48^b^ (± 3.10)	18.32^c^ (± 1.68)	57.81 (< 0.001)
	Colorimeter	Whole‐cookie	51.73^ab^ (± 0.65)	47.12^bc^ (± 2.37)	57.67^a^ (± 4.28)	51.44^ab^ (± 1.87)	40.46^c^ (± 1.79)	19.64 (< 0.001)
	Colorimeter	Selected‐point	56.61^b^ (± 3.13)	56.03^b^ (± 0.14)	65.00^a^ (± 2.10)	57.08^b^ (± 2.20)	49.83^c^ (± 0.50)	22.60 (< 0.001)
	Digital image	Whole‐cookie	33.00^ab^ (± 2.00)	35.33^ab^ (± 5.51)	39.33^a^ (± 4.51)	32.33^ab^ (± 2.52)	28.67^b^ (± 1.53)	3.68 (0.04)
	Digital image	Selected‐point	44.92 (± 5.55)	51.50 (± 3.93)	54.92 (± 2.92)	48.83 (± 2.76)	43.33 (± 5.56)	3.60 (0.05)
	Computer vision	Whole‐cookie	37.40^ab^ (± 1.80)	38.89^ab^ (± 2.84)	44.19^a^ (± 2.12)	38.40^ab^ (± 2.22)	34.71^b^ (± 3.61)	5.32 (0.01)
	Computer vision	Selected‐point	43.09 (± 5.99)	50.70 (± 4.80)	54.14 (± 3.24)	48.56 (± 1.94)	42.78 (± 5.46)	3.52 (0.05)
Redness (a*)	Human sensory evaluation		34.16^b^ (± 5.43)	38.95^b^ (± 3.93)	10.48^c^ (± 1.88)	39.56^b^ (± 2.65)	57.91^a^ (± 4.73)	55.73 (< 0.001)
	Colorimeter	Whole‐cookie	9.44^bc^ (± 0.25)	11.35^ab^ (± 1.06)	7.46^c^ (± 1.03)	10.76^ab^ (± 0.78)	12.14^a^ (± 0.73)	14.97 (< 0.001)
	Colorimeter	Selected‐point	9.12^b^ (± 0.44)	8.54^bc^ (± 1.61)	6.58^c^ (± 0.06)	10.48^ab^ (± 0.58)	11.79^a^ (± 0.37)	17.80 (< 0.001)
	Digital image	Whole‐cookie	8.67^ab^ (± 0.58)	8.33^ab^ (± 0.58)	6.33^b^ (± 1.53)	10.00^ab^ (± 1.73)	11.67^a^ (± 2.08)	5.73 (0.01)
	Digital image	Selected‐point	11.75^b^ (± 1.00)	10.25^bc^ (± 2.13)	6.92^c^ (± 0.52)	12.50^b^ (± 1.98)	17.83^a^ (± 0.14)	24.18 (< 0.001)
	Computer vision	Whole‐cookie	8.04^b^ (± 0.78)	7.33^b^ (± 1.65)	4.28^c^ (± 0.51)	9.00^b^ (± 1.15)	12.54^a^ (± 0.30)	26.63 (< 0.001)
	Computer vision	Selected‐point	9.90^b^ (± 1.07)	8.06^bc^ (± 2.19)	5.09^c^ (± 0.42)	10.19^b^ (± 1.90)	15.60^a^ (± 0.22)	22.60 (< 0.001)
Yellowness (b*)	Human sensory evaluation		41.56^bc^ (± 3.42)	48.85^ab^ (± 1.57)	38.60 ^cd^ (± 4.26)	52.04^a^ (± 1.13)	32.08^d^ (± 4.27)	18.51 (< 0.001)
	Colorimeter	Whole‐cookie	25.30^b^ (± 0.79)	30.95^a^ (± 2.66)	22.49^b^ (± 1.30)	30.60^a^ (± 1.47)	30.10^a^ (± 1.04)	14.56 (< 0.001)
	Colorimeter	Selected‐point	25.96^bc^ (± 1.00)	30.30^ab^ (± 3.10)	25.11^c^ (± 0.72)	33.42^a^ (± 2.04)	29.66^abc^ (± 0.14)	11.24 (0.001)
	Digital image	Whole‐cookie	22.33 (± 1.15)	26.00 (± 1.73)	21.67 (± 3.51)	25.00 (± 1.00)	22.67 (± 1.20)	2.37 (0.12)
	Digital image	Selected‐point	29.17^bc^ (± 3.50)	36.33^a^ (± 1.28)	26.33^c^ (± 0.76)	38.08^a^ (± 1.20)	33.83^ab^ (± 2.67)	13.20 (< 0.001)
	Computer vision	Whole‐cookie	22.63^bc^ (± 1.81)	26.58^a^ (± 1.13)	19.37^c^ (± 0.24)	28.29^a^ (± 1.86)	25.02^ab^ (± 1.31)	18.66 (< 0.001)
	Computer vision	Selected‐point	28.08^bc^ (± 4.11)	35.38^a^ (± 0.77)	25.44^c^ (± 0.73)	37.82^a^ (± 1.18)	33.07^ab^ (± 2.82)	14.34 (< 0.001)

Mean values with different superscripts within a row indicate a significant difference among brands at *p* < 0.05.

For redness (a*), significant brand differences were observed across methods, including sensory evaluation (*p* < 0.05). Brand E was consistently the reddest, whereas Brand C was the least red. *Post hoc* comparisons from whole‐cookie computer vision analysis most closely paralleled the sensory results: Brand E > Brand D = Brand B = Brand A > Brand C.

For yellowness (b*), sensory evaluation indicated Brand D was more yellow than Brands A, C, and E, and did not differ significantly from Brand B. Instrumental colorimetry showed a related, although not identical, pattern, with Brands D, B, and E not differing significantly from one another, although Brand D exceeded Brands A and C. Digital image analysis under whole‐cookie sampling did not detect significant brand differences in b*. Computer vision analyses under both sampling conditions indicated Brands D and B had higher b* values than Brands A and C, whereas neither differed significantly from Brand E.

### 3.3 Similarity of Sensory and Analytical Multivariate Configurations

**FIGURE 4:**
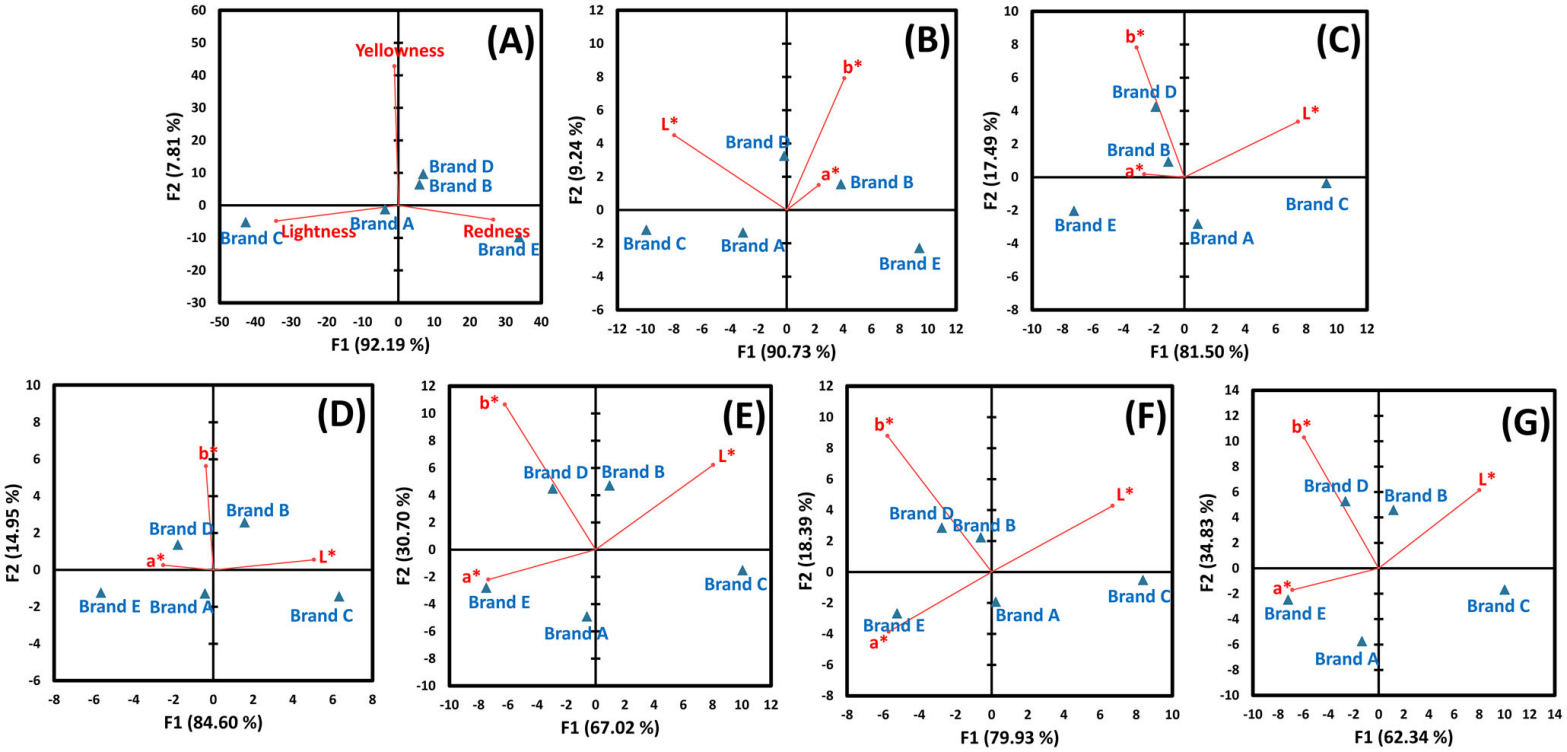
Principal component analysis (PCA) biplots based on lightness (L*), redness (a*), and yellowness (b*) values for five brands of chocolate chip cookies derived from human sensory evaluation (A); instrumental colorimetry under whole‐cookie (B) and selected‐point (C) sampling conditions; digital image analysis under whole‐cookie (D) and selected‐point (E) sampling conditions; and computer vision‐based analysis under whole‐cookie (F) and selected‐point (G) sampling conditions.

Figure 4 presents PCA biplots for the sensory and analytical datasets. In the sensory data and instrumental colorimetry under whole‐cookie sampling, the first principal component (F1) explained more than 90% of the variance and was primarily associated with lightness (L*) and redness (a*), whereas the second component (F2), associated with yellowness (b*), explained less than 10%. Instrumental colorimetry showed a similar structure under both whole‐cookie sampling (F1: 90.73%; F2: 9.24%) and selected‐point sampling (F1: 81.50%; F2: 17.49%). For digital image analysis, F1 explained 84.60% of the variance under whole‐cookie sampling, whereas the variance was more evenly distributed under selected‐point sampling (F1: 67.02%; F2: 30.70%). Computer vision analysis showed a comparable pattern, with a larger contribution of F2 under selected‐point sampling (whole‐cookie: F1 79.93%, F2 18.39%; selected‐point: F1 62.34%, F2 34.83%). Across datasets, brand separation was driven primarily by L* and a*, whereas Brands B and D were more strongly associated with b*. RV coefficients indicated significant similarity between the sensory and analytical matrices, with the strongest alignment observed for selected‐point instrumental colorimetry and whole‐cookie computer vision analysis (both, *p* < 0.001; Table 2).

**TABLE 2:** RV coefficients between principal component analysis (PCA) matrices derived from human sensory evaluation and analytical methods.

Analytical methods	Sampling conditions	Human sensory evaluation		
		RV coefficient	Standardized RV coefficient	*p* value
Instrumental colorimetry	Whole‐cookie	0.93	2.69	0.01
	Selected‐point	0.98	2.74	<0.001
Digital image analysis	Whole‐cookie	0.90	2.48	0.03
	Selected‐point	0.88	2.51	0.02
Computer vision‐based analysis	Whole‐cookie	0.96	2.53	<0.001
	Selected‐point	0.84	2.37	0.02

**FIGURE 5:**
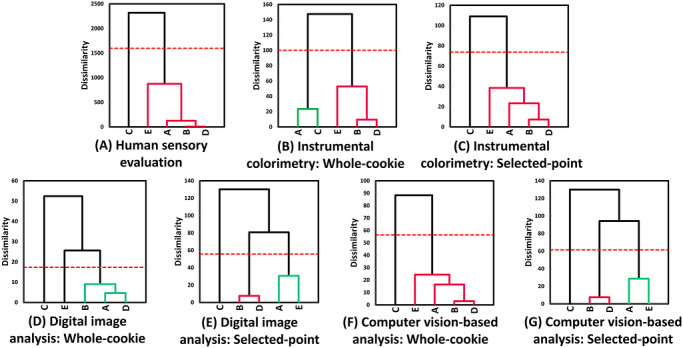
Agglomerative hierarchical clustering (AHC) dendrograms for five brands of chocolate chip cookies based on color parameters obtained from human sensory evaluation (A); instrumental colorimetry under whole‐cookie (B) and selected‐point (C) sampling conditions; digital image analysis under whole‐cookie (D) and selected‐point (E) sampling conditions, and computer vision‐based analysis under whole‐cookie (F) and selected‐point (G) sampling conditions.

AHC dendrograms (Figure 5) were consistent with the multivariate results. Selected‐point instrumental colorimetry and whole‐cookie computer vision analysis reproduced the sensory clustering pattern by separating Brand C (Cluster 1) from Brands A, B, D, and E (Cluster 2). Whole‐cookie instrumental colorimetry produced a different partition, grouping Brands A and C and Brands B, D, and E together. Digital image analysis under whole‐cookie sampling produced three clusters: Cluster 1 (Brand C), Cluster 2 (Brand E), and Cluster 3 (Brands A, B, and D).

### 3.4 Predictive Models for Human Sensory Ratings

Table 3 summarizes the best‐performing models for predicting sensory ratings from analytical parameters (see Tables S1–S3 for full model comparisons). Selected‐point instrumental colorimetry produced the strongest predictions for perceived lightness; a quintic polynomial provided the best fit (*R* ^2^ = 0.93, RMSE = 6.73, AICc = 122.08, BIC = 111.04). For perceived redness, whole‐cookie computer vision analysis yielded the most accurate predictions (*R* ^2^ = 0.92, RMSE = 5.29, AICc = 104.56, BIC = 101.43), with a cubic model consistently ranking highest. For perceived yellowness, selected‐point instrumental colorimetry again showed the best performance (*R* ^2^ = 0.87, RMSE = 3.55, AICc = 102.90, BIC = 91.85), with a quintic polynomial providing the best fit. Overall, under the conditions of the present study, selected‐point instrumental colorimetry emerged as the best‐performing method across color dimensions, followed by whole‐cookie computer vision analysis.

**TABLE 3:** Model‐comparison metrices for predicting human perception from analytical methods and test conditions for lightness, redness, and yellowness, using linear and non‐linear regressions.

Analytical methods	Sampling conditions	Human perception of lightness					Human perception of redness					Human perception of yellowness				
		Models	*R* ^2^	RMSE	AIC_C_	BIC	Models	*R* ^2^	RMSE	AIC_C_	BIC	Models	*R* ^2^	RMSE	AIC_C_	BIC
**Instrumental colorimetry**	Whole‐cookie	Linear	0.74	10.93	120.38	120.31	Linear	0.78	7.82	110.31	110.25	Linear	0.14	7.59	109.39	109.33
	Selected‐point	Quintic	0.93	6.73	122.08	111.04	Linear	0.79	7.59	109.43	109.37	Quintic	0.87	3.55	102.90	91.85
**Digital image analysis**	Whole‐cookie	Linear	0.56	14.27	128.35	128.29	Linear	0.62	10.33	118.66	118.60	Linear	0.25	7.08	107.33	107.28
	Selected‐point	Quadratic	0.51	15.63	133.70	132.53	Cubic	0.90	5.75	107.12	103.99	Quadratic	0.34	6.94	109.32	108.15
**Computer vision‐based analysis**	Whole‐cookie	Linear	0.63	13.00	125.56	125.51	Cubic	0.92	5.29	104.56	101.43	Quadratic	0.37	6.74	108.48	107.32
	Selected‐point	Quadratic	0.49	15.87	134.15	132.99	Cubic	0.91	5.59	106.21	103.08	Quadratic	0.36	6.82	108.81	107.64

RMSE, AIC_C_, and BIC refer to the root mean square error, the corrected Akaike information criterion, and the Bayesian information criterion, respectively.

## 4 Discussion

This study compared instrumental colorimetry, digital image analysis, and computer vision‐based analysis for quantifying the color attributes of chocolate chip cookies and predicting consumer color perception. Across methods, both sensory and analytical approaches reliably discriminated among brands, indicating that the cookie set exhibited a broad range of appearance differences. Importantly, the study extends previous comparisons of instrumental and image‐based methods in foods (Kane et al. 2003; Yam and Papadakis 2004; Afshari‐Jouybari and Farahnaky 2011; Sun et al. 2016; Goñi and Salvadori 2017; Tomasevic et al. 2019; Sabat et al. 2024) by directly evaluating which instrumental strategy most closely corresponds to human perception and by explicitly testing the effects of sampling condition (selected‐point vs. whole‐cookie averaging).

### 4.1 Sampling Condition Systematically Altered Measured Color, Especially Lightness

Across instrumental colorimetry, Photoshop‐based digital image analysis, and OpenCV‐based computer vision, selected‐point sampling generally yielded higher L* values (lighter) than whole‐cookie averaging. This pattern is consistent with a fundamental property of visually heterogeneous foods: Whole‐surface or whole‐sample averages integrate both lighter dough regions and darker features, such as chocolate chips, localized browning, and edge darkening, thereby lowering the mean L* relative to measurements restricted to smaller regions that may not fully capture the darkest areas (Pedreschi et al. 2006). Chocolate chip cookies are visually complex and spatially non‐uniform (Frauenhoffer et al. 2025); accordingly, the magnitude and direction of sampling effects are expected to depend on chip coverage, browning gradients, and surface texture.

For instrumental colorimetry specifically, the “whole‐cookie” condition in this study involved homogenization by crushing/smashing, which differs conceptually from “whole‐cookie” in imaging (surface averaging; Figure 1). Homogenization may further reduce L* values by dispersing dark chocolate materials into the dough matrix and by altering surface scattering properties relative to those of an intact cookie surface. This distinction is not necessarily a limitation; rather, it highlights that “whole‐cookie” can represent two different measurement concepts: a bulk composite (homogenized) approach versus a surface‐integrated approach. The observed differences, therefore, emphasize the importance of explicitly defining the sampling frame when comparing analytical modalities and interpreting their correspondence with perception.

### 4.2 Analytical Method Conditions Affected Color Parameters of Cookies

Even when targeting the same conceptual sampling condition, systematic differences were observed among methods. Such offsets are common when comparing colorimeters with image‐derived measurements because the underlying technologies differ in illumination, viewing geometry, and the way they integrate spatial information (Chae 2022).

Colorimeters typically use standardized optics and illumination to measure a defined spot size and return CIELAB values directly. In contrast, imaging approaches acquire RGB values that are influenced by camera sensor characteristics, lens geometry, exposure, white balance, and shading (Wang et al. 2024). Converting RGB values to CIELAB coordinates introduces additional dependence on calibration assumptions, including illuminant, color profile, and conversion algorithms. These sources of variability have been widely recognized in food imaging and computer vision applications (Afshari‐Jouybari and Farahnaky 2011; Sun et al. 2016; Wang et al. 2024). Consequently, agreement between imaging and colorimetry is often product‐ and setup‐dependent, and the direction and magnitude of bias may differ across studies (Kane et al. 2003; Tomasevic et al. 2019).

Within this context, the present findings suggest that selected‐point instrumental colorimetry provided the most stable approximation of perceived lightness (L*) and yellowness (b*), whereas whole‐cookie computer vision performed comparatively better for predicting perceived redness (a*). One plausible interpretation is that perceived redness in cookies is driven more strongly by broad surface cues, such as overall browning tone and Maillard reaction–related color development, which are effectively captured by whole‐surface pixel averaging. In contrast, perceived lightness and yellowness may depend more heavily on local contrast and on the specific regions that consumers use as visual reference points.

### 4.3 Human Color Judgments Reflect Integrated Appearance Cues of Cookies

A key contribution of this study is the direct comparison between analytical measurements and human perception. Although CIELAB was designed to approximate perceptual uniformity, sensory judgments of “lightness,” “redness,” and “yellowness” in complex foods may not map directly onto instrumental L*, a*, and b* values. Consumers do not “sample” the surface in the same way as an instrument. Instead, they integrate multiple visual cues, including contrast, spatial distribution of dark inclusions (e.g., chocolate chips), gloss, visual texture, and edge browning, all of which may influence perceived color magnitude even when mean colorimetric values are smaller. This issue is particularly relevant for chocolate chip cookies, in which the chips function as salient dark features that may bias perceived darkness beyond what would be predicted by a simple average.

The divergence observed for b* across approaches is consistent with the possibility that participants interpreted “yellowness” as a broader appearance attribute related to “golden‐brown” impressions rather than as a strict yellow‐blue axis. Under such interpretations, instrumental b* may not fully capture the perceptual construct used by participants. Similar discrepancies between sensory appearance terms and instrumental indices have been reported in other foods, especially when surface heterogeneity is high (Segnini et al. 1999; Quevedo et al. 2010). In chocolate chip cookies, Kane et al. (2003) reported stronger correspondence between sensory ratings and instrumental L* and a* values than with b*, further illustrating the difficulty of linking b* to perceived “yellowness” in this product category. Taken together, these considerations support the value of using multivariate similarity metrics (e.g., RV coefficients) and clustering patterns, rather than relying solely on univariate correlations when evaluating agreement between analytical color measurements and perception.

### 4.4 Multivariate Agreement Suggests Two Practical “Best Use” Scenarios of Analytical Methods

The PCA, RV coefficients, and AHC results converged on a consistent interpretation. Selected‐point instrumental colorimetry and whole‐cookie computer vision most closely resembled the sensory configuration. This suggests two practically useful analytical pathways. First, selected‐point colorimetry appears to provide the closest overall proxy for consumer judgments, particularly for perceived lightness (L*) and yellowness (b*). Its controlled optics and direct CIELAB output likely reduce variability and improve comparability across sessions and laboratories. Second, whole‐cookie computer vision performed competitively and produced the strongest predictive performance for perceived redness. This finding is notable because imaging‐based methods are more scalable and accessible than colorimeters in home‐based or small‐scale production settings. Computer vision also enables full‐surface characterization and may better capture the visual “gestalt” consumers perceive when viewing heterogeneous products (Wagemans et al. 2012).

Importantly, this study's findings do not indicate that one method is universally superior. Rather, method suitability depends on the perceptual attributes of interest and on whether the goal is localized standardization, as with instrumental colorimetry, or surface‐integrated appearance representation, as with imaging and computer vision.

### 4.5 Predictive Modeling Supports Non‐Linear Relationships Between Instrumental Measures and Perception

Model comparisons indicated that polynomial regressions frequently outperformed simpler models for specific parameter–method combinations. This is plausible because the relationship between instrumental indices and perception may be non‐linear, especially when perceptual responses compress at extremes, when contrast effects are present, or when spatial heterogeneity alters the way that observers weigh different regions of the surface. Previous work has similarly shown that non‐linear and machine‐learning models can be effective for predicting color‐related outcomes in foods (León et al. 2006; Wang et al. 2024), and that model selection should be guided by predictive error and information criteria rather than complexity alone (Fahrmeir et al. 2021). From an applied standpoint, the present findings suggest that relatively interpretable non‐linear models (e.g., low‐order polynomials) may offer a practical compromise between predictive accuracy and implementation simplicity in industrial or small‐scale workflows.

### 4.6 Implications, Limitations, and Future Studies

These findings have several practical implications for the food industry. First, they can help manufacturers select the most appropriate color measurement method according to their specific objective. In the present study, selected‐point instrumental colorimetry most closely reflected perceived lightness and yellowness of chocolate chip cookies, whereas whole‐cookie computer vision provided the best predictions of perceived redness. These results offer a more evidence‐based foundation for method selection rather than assuming that all color measurement approaches are interchangeable. Second, the results demonstrated that the sampling condition is a critical factor in cookie color measurement. Color values varied not only by analytical method but also by sampling strategy, indicating that method choice alone is insufficient without careful consideration of how the sample is represented. Food industry professionals should therefore select sampling conditions that align with both their product characteristics and their measurement objectives. Third, the findings suggest that computer vision may serve as a scalable and accessible tool for food applications. Because imaging methods can evaluate the entire visible surface and may better capture the integrated appearance of heterogeneous foods such as chocolate chip cookies, they may be useful for rapid screening of product appearance during processing and development, as well as for small‐scale manufacturers that do not have access to more expensive colorimeters.

Several limitations should be considered when interpreting these findings. First, although a controlled light box was used, image‐derived color values remain sensitive to camera settings, illumination conditions, and color management (Wang et al. 2024). Incorporating standardized color targets and clearly reporting calibration workflows would further improve reproducibility and inter‐laboratory comparability (Afshari‐Jouybari and Farahnaky 2011; Sun et al. 2016). Second, participants rated “yellowness” and “redness” using line scales intended to mimic CIELAB axes, but sensory interpretations of these terms may differ from strict colorimetric definitions. Future studies could test alternative descriptors (e.g., golden color or brownness) or include anchors to better align sensory scales with the intended constructs. Third, the number of brands included in the multivariate analyses was limited, particularly for the PCA‐based visualizations. Accordingly, the proportion of variance explained by the first principal components should be interpreted cautiously and not taken as evidence of broader robustness. Future studies, including a larger and more diverse set of brands, would help strengthen the stability of the multivariate patterns. Finally, because the regression models were developed using a small dataset, they should be considered exploratory rather than definitive predictive functions. Validation with an independent set of cookie samples would be necessary to confirm robustness beyond the current dataset, consistent with best practices in predictive modeling (Afshari‐Jouybari and Farahnaky 2011).

## 5 Conclusions

Taken together, the results indicate that selected‐point instrumental colorimetry was the most consistent approach for approximating consumer perception across color dimensions in chocolate chip cookies, while whole‐surface computer vision offered an effective and scalable alternative, particularly for predicting perceived redness. The combined evidence from multivariate similarity analysis (RV coefficient), clustering (AHC), and predictive modeling shows that both measurement method and sampling condition influence the degree of correspondence with human perception. These findings provide practical guidance for selecting color evaluation workflows in contexts ranging from industrial quality control to small‐scale food production.

## Author Contributions

**Natalia Calderón**: conceptualization, methodology, investigation, formal analysis, writing – original draft. **Eniola Ola**: conceptualization, methodology, investigation, writing – review and editing. **Han‐Seok Seo**: conceptualization, methodology, investigation, funding acquisition, visualization, writing – original draft, writing – review and editing, project administration, resources.

## Funding

This study was based upon work that is supported, in part, by the United States Department of Agriculture National Institute of Food and Agriculture Hatch Act funding (7001030) to H.‐S.S.

## Conflicts of Interest

The authors declare no conflicts of interest.

## Supporting information




**Supplementary Tables**: jfds71133‐sup‐0001‐tablesS1‐S3.pdf
