# Supplementary material for: Evaluating the Color of Chocolate Chip Cookies: Comparative Performance of Instrumental Colorimetry, Digital Image Analysis, and Computer Vision Relative to Human Perception
Source: J Food Sci. 2026 May 11;91:e71133. doi: 10.1111/1750-3841.71133 (PMC13158829; doi:10.1111/1750-3841.71133)
Supplement: Supplementary file 1 — Supplementary Tables: jfds71133‐sup‐0001‐tablesS1‐S3.pdf [file JFDS-91-0-s001.pdf]

Supplementary Table S1. List of model metrics for predicting human perception of lightness from analytical methods and test conditions, using linear and non-linear regressions

Analytical methods	Sampling conditions	Models	R^2	RMSE	AIC_C	BIC
Instrumental colorimetry	Whole-cookie	Linear	0.74	10.93	120.38	120.31
		Power	0.76	10.85	122.74	121.58
		Quadratic	0.76	10.88	122.81	121.65
		Cubic	0.77	11.17	126.99	123.86
		Quartic	0.78	11.47	132.17	125.92
		Quintic	0.79	11.82	139.00	127.96
	Selected-point	Linear	0.81	9.20	115.18	115.12
		Power	0.82	9.27	118.02	116.86
		Quadratic	0.83	9.29	118.10	116.94
		Cubic	0.91	7.40	119.02	112.77
		Quartic	0.85	9.13	120.93	117.81
		Quintic	0.93	6.73	122.08	111.04
Digital image analysis	Whole-cookie	Linear	0.56	14.27	128.35	128.29
		Power	0.59	14.35	131.14	129.97
		Quadratic	0.59	14.42	131.28	130.11
		Cubic	0.59	14.82	135.47	132.34
		Quartic	0.63	14.80	139.82	133.57
		Quintic	0.65	15.18	146.50	135.45
	Selected-point	Linear	0.36	17.13	133.82	133.77
		Power	0.41	17.13	136.44	135.28
		Quadratic	0.51	15.63	133.70	132.53
		Cubic	0.51	16.32	138.36	135.23
		Quartic	0.55	16.33	142.77	136,52
		Quintic	0.60	16.29	148.61	137.57
Computer vision-based analysis	Whole-cookie	Linear	0.63	13.00	125.56	125.51
		Power	0.67	12.87	127.88	126.71
		Quadratic	0.67	12.91	127.96	126.79
		Cubic	0.67	13.38	132.40	129.27
		Quartic	0.76	12.06	133.70	127.45
		Quintic	0.78	12.15	139.83	128.79
	Selected-point	Linear	0.33	17.62	134.69	134.63
		Power	0.38	17.62	137.30	136.13
		Quadratic	0.49	15.87	134.15	132.99
		Cubic	0.50	16.49	138.67	135.54
		Quartic	0.54	16.65	143.36	137.11
		Quintic	0.56	17.17	150.22	139.17

RMSE, AIC_C, and BIC refer to the root mean square error, the corrected Akaike information criterion, and the Bayesian information criterion, respectively.

Supplementary Table S2. List of model metrics for predicting human perception of redness from analytical methods and test conditions, using linear and non-linear regressions

Analytical methods	Sampling conditions	Models	R^2	RMSE	AIC_C	BIC
**Instrumental colorimetry**	**Whole-cookie**	Linear	0.78	7.82	110.31	110.25
		Power	0.78	8.07	113.87	112.71
		Quadratic	0.79	8.05	113.79	112.62
		Cubic	0.79	8.24	117.84	114.71
		Quartic	0.81	8.36	122.70	116.44
		Quintic	0.81	8.71	129.84	118.80
	**Selected-point**	Linear	0.79	7.59	109.43	109.37
		Power	0.79	7.89	113.18	112.02
		Quadratic	0.79	7.89	113.18	112.00
		Cubic	0.80	8.04	117.10	113.98
		Quartic	0.82	8.08	121.67	115.42
		Quintic	0.83	8.36	128.61	117.57
**Digital image analysis**	**Whole-cookie**	Linear	0.62	10.33	118.66	118.60
		Power	0.66	10.15	120.75	119.59
		Quadratic	0.66	10.06	120.50	119.34
		Cubic	0.66	10.51	125.16	122.04
		Quartic	0.70	10.45	129.39	123.14
		Quintic	0.73	10.44	135.29	124.24
	**Selected-point**	Linear	0.81	7.31	108.27	108.21
		Power	0.84	6.85	108.96	107.79
		Quadratic	0.83	7.13	110.13	108.97
		Cubic	0.90	5.76	107.12	103.99
		Quartic	0.90	6.04	112.95	106.69
		Quintic	0.90	6.36	120.41	109.37
**Computer vision-based analysis**	**Whole-cookie**	Linear	0.86	6.14	103.05	102.99
		Power	0.89	5.81	103.99	102.82
		Quadratic	0.88	5.99	104.93	103.76
		Cubic	0.92	5.29	104.56	101.43
		Quartic	0.92	5.55	110.39	104.13
		Quintic	0.92	5.56	116.38	105.33
	**Selected-point**	Linear	0.79	7.57	109.33	109.28
		Power	0.83	7.16	110.28	109.11
		Quadratic	0.81	7.51	111.71	110.54
		Cubic	0.91	5.59	106.21	103.08
		Quartic	0.91	5.80	111.75	105.50
		Quintic	0.91	6.07	119.01	107.97

RMSE, AIC_C, and BIC refer to the root mean square error, the corrected Akaike information criterion, and the Bayesian information criterion, respectively.

Supplementary Table S3. List of model metrics for predicting human perception of yellowness from analytical methods and test conditions, using linear and non-linear regressions

Analytical methods	Sampling conditions	Models	R^2	RMSE	AIC_C	BIC
**Instrumental colorimetry**	**Whole-cookie**	Linear	0.14	7.59	109.39	109.33
		Power	0.14	7.89	113.21	112.04
		Quadratic	0.14	7.89	113.21	112.04
		Cubic	0.14	8.23	117.82	114.69
		Quartic	0.15	8.63	123.63	117.38
		Quintic	0.16	9.00	130.84	119.79
	**Selected-point**	Linear	0.25	7.08	107.31	107.25
		Power	0.27	7.24	110.62	109.45
		Quadratic	0.31	7.03	109.73	108.56
		Cubic	0.34	7.21	113.87	110.74
		Quartic	0.76	4.55	104.43	98.18
		Quintic	0.87	3.55	102.90	91.85
**Digital image analysis**	**Whole-cookie**	Linear	0.25	7.08	107.33	107.28
		Power	0.26	7.32	110.93	109.76
		Quadratic	0.26	7.31	110.89	109.72
		Cubic	0.27	7.59	115.38	112.25
		Quartic	0.30	7.79	120.59	114.33
		Quintic	0.36	7.88	126.85	115.80
	**Selected-point**	Linear	0.25	7.09	107.38	107.33
		Power	0.28	7.22	110.52	109.35
		Quadratic	0.34	6.94	109.32	108.15
		Cubic	0.34	7.24	113.99	110.86
		Quartic	0.34	7.60	119.82	113.57
		Quintic	0.34	7.97	127.17	116.13
**Computer vision-based analysis**	**Whole-cookie**	Linear	0.29	6.90	106.53	106.47
		Power	0.33	6.97	109.48	108.31
		Quadratic	0.37	6.74	108.48	107.32
		Cubic	0.37	7.04	113.14	110.01
		Quartic	0.38	7.33	118.74	112.48
		Quintic	0.40	7.65	125.96	114.91
	**Selected-point**	Linear	0.26	7.03	107.13	107.07
		Power	0.30	7.14	110.19	109.02
		Quadratic	0.36	6.82	108.81	107.64
		Cubic	0.37	7.09	113.33	110.21
		Quartic	0.40	7.23	118.34	112.09
		Quintic	0.41	7.50	125.35	114.31

RMSE, AIC_C, and BIC refer to the root mean square error, the corrected Akaike information criterion, and the Bayesian information criterion, respectively.
